# The Use of Digital Technologies to Support Vaccination Programmes in Europe: State of the Art and Best Practices from Experts’ Interviews

**DOI:** 10.3390/vaccines9101126

**Published:** 2021-10-03

**Authors:** Anna Odone, Vincenza Gianfredi, Sebastiano Sorbello, Michele Capraro, Beatrice Frascella, Giacomo Pietro Vigezzi, Carlo Signorelli

**Affiliations:** 1 Department of Public Health, Experimental and Forensic Medicine, University of Pavia, 27100 Pavia, Italy; sebastianosorbello@hsph.harvard.edu; 2 School of Medicine, Vita-Salute San Raffaele University, Via Olgettina 58, 20132 Milan, Italy; gianfredi.vincenza@hsr.it (V.G.); capraro.michele@hsr.it (M.C.); frascella.beatrice@hsr.it (B.F.); vigezzi.giacomopietro@hsr.it (G.P.V.); signorelli.carlo@hsr.it (C.S.)

**Keywords:** vaccination, immunisation programmes, digitalisation, information and communication technology, Europe, public health practice

## Abstract

Digitalisation offers great potential to improve vaccine uptake, supporting the need for effective life-course immunisation services. We conducted semi-structured in-depth interviews with public health experts from 10 Western European countries (Germany, Greece, Italy, Luxembourg, Malta, the Netherlands, Norway, Poland, Portugal, and the United Kingdom) to assess the current level of digitalisation in immunisation programmes and retrieve data on interventions and best practices. Interviews were performed using an ad hoc questionnaire, piloted on a sample of national experts. We report a mixed level of digital technologies deployment within vaccination services across Europe: Some countries are currently developing eHealth strategies, while others have already put in place robust programmes. Institutional websites, educational videos, and electronic immunisation records are the most frequently adopted digital tools. Webinars and dashboards represent valuable resources to train and support healthcare professionals in immunisation services organisation. Text messages, email-based communication, and smartphone apps use is scattered across Europe. The main reported barrier to the implementation of digital-based programmes is the lack of resources and shared standards. Our study offers a comprehensive picture of the European context and shows the need for robust collaboration between states and international institutions to share best practices and inform the planning of digital intervention models with the aim of countering vaccine hesitancy and increasing vaccine uptake.

## 1. Introduction

Vaccines are widely considered one of the most efficient primary prevention tools for promoting individual and public health, responsible for drastic reductions in the burden of infectious diseases and associated mortality worldwide [1,2]. Nevertheless, vaccination coverage is still suboptimal, this having marked the resurgence of vaccine-preventable disease outbreaks in recent times [3,4,5]. For example, in the past three years, seven countries, four in Europe, have lost their measles elimination status [6]. The European Vaccine Action Plan 2015–2020 has just ended its course, and vaccination coverage is still below the predefined herd immunity thresholds [7]. Consequently, the new European Immunisation Agenda 2030 was launched to address inequalities in vaccination coverage between and within countries [8]. The main objectives of this flagship initiative are to systematically tackle issues in the supply and delivery of vaccines, including community demand and acceptance, and to fight vaccine hesitancy and the spread of misinformation.

During the 71st World Health Assembly [9] in 2018, country delegates resolved to boost the spread of digital technologies and the development of new ones for healthcare, including immunisation. These might help to counter the falling vaccination rates and dangerous under-immunisation pockets, that the COVID-19 pandemic contributed to aggravate [10,11] and whose determinants include low public trust in vaccines, constraints on affordability or access, and an emerging crisis in vaccine hesitancy [12,13]. As declared by the World Health Organization (WHO), eHealth and the field of digital health, defined as “cost-effective and safe use of information and communication technologies in support of health and health-related areas” [8,14], represent an important resource to achieve this goal. This is particularly true because the field of digital health has flourished in recent years, revolutionising the processes of gathering, spreading, and utilising health information among healthcare providers, citizens, and mass media [7]. Thanks to their variety and continuously evolving nature, digital technologies can be employed to support vaccination campaigns in critical phases [15]: for instance, to fight misinformation and hesitancy in communication campaigns [9,16,17] and the implementation of vaccination programmes, as well as to monitor vaccination coverage [18]. In recent times, vaccination programmes have been including digital technologies components, with promising results. However, their use and effectiveness are far from being comprehensively explored or quantified [19,20,21,22,23,24,25,26].

On these premises, we designed and coordinated the EUrope Vaccines Information and communication technologies (ICT) Strategies (EUVIS) project to analyse the evidence on the effectiveness of vaccination services employing digital technologies in Europe and across the world, and ultimately inform the planning of effective digital interventions to counter vaccine hesitancy and increase vaccine uptake.

The first phase of the EUVIS project consisted of systematic assessments of digitalisation’s use, effectiveness, and impact in immunisation programmes. Our comprehensive analysis showed that, even though technological innovations have made significant contributions to healthcare, their effectiveness to increase vaccination uptake cannot be taken for granted. The most beneficial effect emerged when digital innovations were tailored to specific characteristics and needs of the target populations. Having identified gaps of knowledge in the field, we conducted a more in-depth analysis of specific digital technologies, with particular reference to email-based immunisation reminders [27] and personal electronic health records (EHR) [28].

On the basis of the evidence gathered from the systematic reviews, the aim of the second part of the EUVIS project, reported in the current study, was to collect original quantitative and qualitative data to comprehensively assess the level of digitalisation of immunisation programmes, best practices, and policies across Europe. 

The study was designed with three main objectives: (i) to provide an updated overview of the level of implementation of digital technologies in the context of immunisation programmes in Europe; (ii) to collect and report best practices of design, planning, implementation, and evaluation of the performance of immunisation campaigns based on digital technologies, in selected European countries; (iii) to perform a qualitative analysis of the information collected to support the planning of immunisation campaigns based on digital technologies in the future. 

## 2. Materials and Methods

Our study settings were European countries, including Germany, Greece, Italy, Luxembourg, Malta, the Netherlands, Norway, Poland, Portugal, and the United Kingdom, representing both Northern, Central, and Southern Europe. We applied both qualitative and quantitative research methods. Country data were compiled through desk reviews of relevant socio-demographic, health systems, and digitalisation data; original data were collected through semi-structured interviews with public health and high-level immunisation experts in Europe.

First, we performed a comprehensive search of institutional and governmental websites of included countries on national e-Health policies and immunisation campaigns based on digital tools. An overview of included countries’ features regarding demographic and socio-economic indicators, vaccination coverage, and indicators of digitalisation is presented in Table 1. We analysed these factors via digitalisation indicators available in the Eurostat online database: household internet access, individuals using the internet, personal digital inclusion, e-government activities via websites, and subjects who have basic or above basic overall digital skills [29].

### Survey Development and Administration

In order to better analyse the level of implementation of digital strategies and optimise the collection of qualitative data, we identified semi-structured interviews with relevant experts in the field as a suitable survey method for the study. This format allowed us to collect data following the structure of an ad-hoc questionnaire as a guideline and, at the same time, enabled respondents to provide additional comments and insights for a more in-depth understanding of the topic covered. 

**Figure 1:**
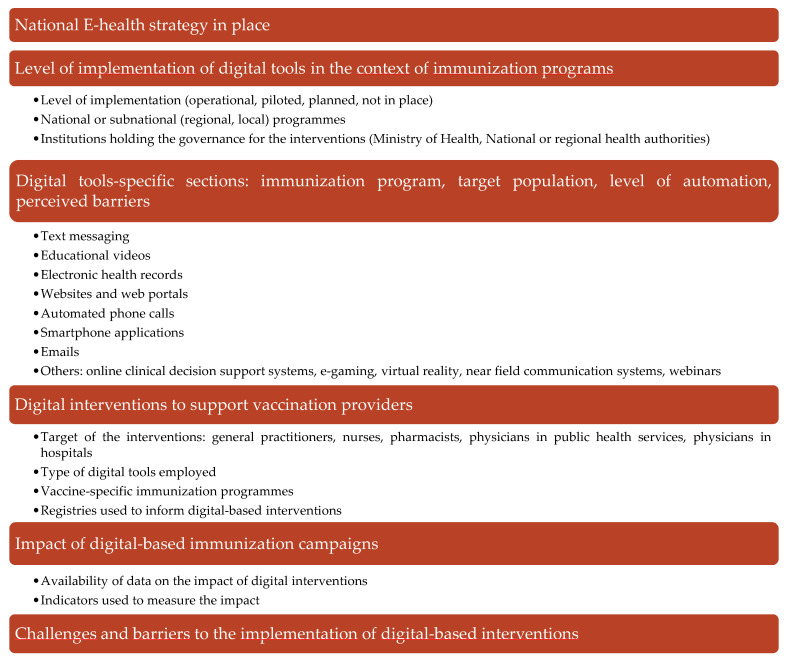
Overview of the structure and content of the interviews.

An overview of the interviews’ questionnaire structure and main features is included in Figure 1. The questionnaire was designed based on EUVIS project part I outputs and updated literature search; it underwent a round of internal validation through an experts’ focus group and an external piloting phase. It comprised six sections and a total of 88 items. The first section assessed the existence and degree of implementation of national and regional e-health strategies in general and specifically regarding immunisation programmes. 

Subsequent sections assessed in detail the level of implementation, target populations, and aim of different ICT-based interventions in the context of immunisation services, including text messaging, educational videos, EHRs, websites, web portals, automated phone calls, smartphone applications, emails, online clinical decision support systems, e-gaming, virtual reality, near field communication systems, and webinars.

A fourth section explored the existence and adoption of ICT-based interventions to support vaccination providers. The last section focused on perceived challenges and barriers faced in implementing and scaling up digitalisation within immunisation.

Semi-structured interviews were conducted with prominent academic figures and public health experts with international and national-level professional, research, and policy experience in vaccines and immunisation.

Vaccination experts for each country were identified with the collaboration of the European Public Health Association (EUPHA) sections of Infectious Diseases Control and Digital Health, the Vaccine European New Integrated Collaboration Effort (VENICE) project Gatekeepers, or through referral by public health figures. 

Each interview lasted on average 1.5 h and was conducted remotely by a team of 2–3 members of the research group. After obtaining permission from the participants, the recordings of the interviews were transcribed for comprehensive information extraction. The interviews were followed by contacts via email, allowing respondents to provide further information and references to analyse the data collected. Data were then extracted quantitatively, qualitatively analysed, and pooled. Results were included in a report and presented by country and by digital tool.

## 3. Results

### 3.1. eHealth Strategies in Europe 

In 2018, the European Commission (EC) adopted a strategy for the digital transformation of healthcare in the Digital Single Market, intending to improve citizen empowerment [30]. This strategy has three priorities: (i) to enable citizens to access their health data across the European Union (EU), (ii) to advance personalised medicine through a shared European infrastructure, and (iii) to implement digital tools for user feedback, person-centred care, and interaction between users and healthcare providers. 

All interviewed experts referred to the European strategy and reported the adoption of national-level strategies for eHealth (electronic health) development in their country, if implemented. Adopted eHealth strategies generally aim at the integration of all information sources involved in the delivery of healthcare via technology-based systems and at the implementation of a fully integrated supply chain, involving high levels of automation and information sharing. These plans are directed by national governments and put in place by national, regional, or local health authorities with the help of dedicated agencies. 

In the Netherlands, the national eHealth strategy encompasses multiple fields. Since 2005, a nationwide EHR has been implemented, linked to the Dutch national infrastructure to exchange data between healthcare providers (called *AORTA*) [31]. The Ministry of Health, Welfare and Sport works in cooperation with the National IT Institute for Healthcare and the Central Information Point for Healthcare Professions to monitor the development of a nationwide eHealth tool. The application of sustainable telemedicine services [31] is of increasing importance. A national Dutch ICT-based intervention is operational under the governance of the Ministry of Health and the National Health Agency. Regional health agencies are also in charge of developing local ICT-based programmes.

In Italy [32], a national eHealth strategy has been operational since 2008, leading the way for the implementation of electronic certificates, EHRs, eHealth initiatives for pharmacies, such as the adhesion to the Europe-wide e-Prescription initiative, and telemedicine services [33].

In Portugal, the Shared Services of the Ministry of Health was co-created in 2010, as a state-owned enterprise, by the Ministry of Health and the Ministry of Finance, with the mission to both develop and provide shared services to all entities of the Portuguese National Health Service (NHS), from local health providers to national authorities [34]. The Portuguese NHS is well integrated with the eHealth strategy since approximately 90% of the health provider entities use digital healthcare solutions and administrative processes [35].

In Germany, the eHealth law came into effect in 2015 and provided a roadmap for implementing digital applications for the healthcare system, shaping the growth of the eHealth sector substantially [35]. Within this legislation, the German government specifically stated that all medical practices and hospitals were expected to be connected to telematic infrastructures by the end of 2018. The fastest-growing sector is telemedicine, and a system of electronic health cards is also active with the primary purpose of managing prescriptions.

In Norway, the Norwegian Directorate of eHealth, a subordinate institution of the Ministry of Health and Care Services, has been active since 2016. The government has launched an ambitious strategy for research and innovation in health and care, resulting in the “Government Action Plan for Implementation of the Health&Care21 Strategy” [36]. This programme aims to promote innovation in healthcare by funding innovative projects promoting awareness-building and learning platforms, increasing the focus on public procurement to drive innovation and national business development in healthcare. 

In the UK, the NHS is undergoing a digital transformation [37], as outlined in the NHS Long Term Plan in January 2019 [38]. This process is under the surveillance of NHS-user eXperience (NHSX), a unit that brings together the Department of Health and Social Care, NHS England, and NHS Improvement. In particular, the Digital Health Transformation Programme [39] aims at transforming child health information services by promoting the use of apps and digital tools to monitor the health status of children and grant caregivers access to this information. One of the objectives of this programme is the implementation of the National Failsafe Service to deliver alerts to parents in case of missed opportunities to vaccinate their children.

In Luxembourg, the development of an eHealth strategy is in the initial phase, and limited data is available on quality, the performance of the healthcare system, health plan information, and patient access to any form of health data [39].

### 3.2. Digital Tools Employed in the Context of Immunisation Programmes

Our study shows that digital tools are already employed in eight considered countries (80%) to support different components of immunisation programmes, and more digital interventions are under development, both at the national and sub-national levels. 

**Figure 2:**
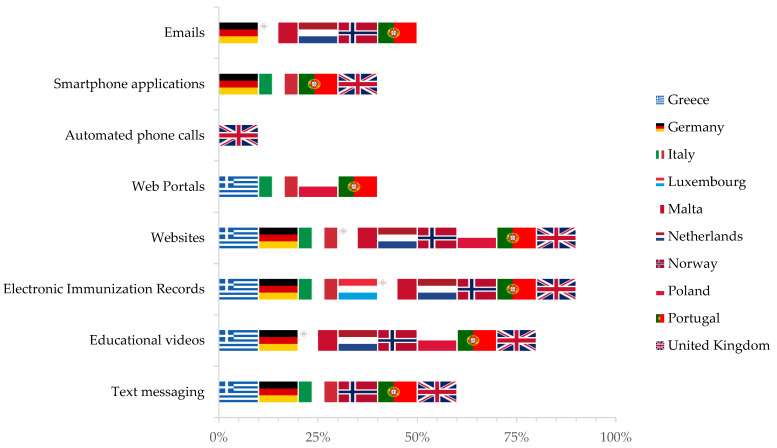
Digital technologies implementation within immunisation programmes, by country (as reported during in-depth interviews with public health and immunization experts).

Institutional websites are the most widespread digital tool (90% of the included countries), followed by educational videos to promote immunisation campaigns (80% of included countries), text messages (six of the included countries, or 60%) and email-based reminder systems (five of the included countries, or 50%). Automatic telephone calls are widely underused in vaccines programmes, implemented only by one country (10%). The systems mentioned above are rarely entirely automated; for instance, only 40% of the email systems send communications automatically (Figure 2).

However, the major efforts across included countries are focused on adopting and developing EHRs. In seven countries (70%), EHRs also include vaccination records. In countries with a double public-private health system, such as Germany, these are implemented mainly by the National Health Agency but also by private providers. In countries with a decentralised health system, such as Italy, substantial differences persist among different regions. 

#### 3.2.1. Electronic Immunisation Records and Immunisation Information Systems (IIS)

EHRs are operational in nine countries (90%) included in this study, but only seven countries (70%) have adopted immunisation records. Such records are often only accessible to healthcare providers to record and monitor the vaccination status of their patients; in some countries, such as Greece, Malta, Portugal and the Netherlands, this platform also allows the delivery of vaccination reminders and recalls [40]. In some regions of Italy, for example, in Lombardy, citizens can activate their EHR, called Fascicolo Sanitario Elettronico, on a voluntary basis and choose who can access their information among healthcare workers on the national territory [41]. These records can include citizens’ vaccination status [41,42]; in addition, an Immunisation Information System (IIS), called Anagrafe vaccinale nazionale, based on the national lifetime immunisation schedule [43], has been operational since 2018 [44]. It is monitored by the regional health authorities and used to evaluate national immunisation coverage and the performance of immunisation campaigns [45]. In Norway, EHRs are also mandatory to record vaccinations provided but not included in the National Immunisation Plan. The interoperability between the EHRs and the IIS permits the monitoring of individual vaccination status by vaccination providers and the estimation of vaccination coverage in the population by public health authorities. 

In the UK, EHRs are operational and include immunisation information regarding the general population. Although the system delivers reminders and recalls, interoperability is still limited, as it can be accessed only by vaccination providers or, depending on the information technology company, by commissioners and public health authorities.

In Luxembourg, where immunisation coverage is already high, the effort is focused on implementing an electronic vaccination registry to link each vaccine administration to a virtual stock in the vaccination centre, to facilitate procurement and supply of sufficient doses of the recommended vaccines. 

#### 3.2.2. Text Messages and Email-Based Systems

The use of text messages in immunisation campaigns was reported in 60% of the countries (six countries: Greece, Germany, Italy, Norway, Portugal, United Kingdom), mainly to deliver reminders and recalls about vaccination deadlines and appointments. The target population for text messages reminders usually includes parents of children, followed by adolescents and young women in the context of HPV vaccination campaigns. 

In Greece, paediatricians and primary care centres can send text messages to parents of children with a tool customising the content of the text, while in the United Kingdom, many companies offer automated systems to deliver text messages, both one-way and two-way (allowing patients to reply and interact) [46]. In Norway, text messages deliver educational content, particularly in two programmes: HPV vaccination for young women and influenza vaccination for the elderly [46]. 

With reference to emailing, automated email systems were described only in 50% of cases (five countries: Germany, Malta, the Netherlands, Norway, Portugal) and employed to deliver reminders and recalls for immunisation. This system is fully automated in only 20% of cases (two countries). In Portugal, the system is semi-automated, and the target population is selected manually by local health centre staff through a database, while the emails are then automatically sent.

#### 3.2.3. Smartphone Applications

The use of smartphone applications in immunisation programmes is modest (40% of countries: Germany, Italy, Portugal, United Kingdom) and heterogeneous in Europe. In most cases, apps are realised and managed by private healthcare companies. For example, in Germany, some apps, such as Vivy^TM^ Health, help keep track of past and upcoming vaccinations by creating a digital vaccination card [47]. In some cases, national or regional institutions have produced smartphone applications to trace healthcare information. For instance, in Italy, the app Salutile Vaccinazioni [47], recommended by the Lombardy region, is linked to local EHRs and allows information retrieval on the user’s immunisation status. 

A national smartphone application is also available in Portugal and the UK. The Portuguese application allows the delivery of educational content, reminders and recalls, and monitoring of vaccination status [48]. In particular, this app contains the list of the various vaccines indicated by the Portuguese National Immunisation Plan for each age group, as well as the recommended doses. Moreover, this app acknowledges users to record the intake of vaccines and be notified whenever a dose date is approaching [42]. The eRedbook is operational in the UK, a virtual version of the Redbook, a booklet that holds children’s growth charts and health records [47]. Advantages of the electronic Redbook are the possibility of receiving health-related guidance tailored to the child’s age and information on immunisation programmes. Interactive charts are available to keep track of the child’s growth. Through the app, parents can share records with other carers. An additional feature of the eRedbook, that is being piloted, is connecting it to the EHRs. In doing so, information on vaccination added by parents to eRedbook will be transferred to the EHRs and subsequently become available to public health authorities and aggregated into performance dashboards.

#### 3.2.4. Educational Videos, Websites, and Portals

Audio-visual material is employed in 80% of included countries to broadcast information campaigns on the benefits of immunisation (eight countries: Greece, Germany, Malta, the Netherlands, Norway, Poland, Portugal, the United Kingdom). These are often initiatives of the ministries of health. Sometimes, paediatrics and geriatrics scientific societies produce video material on specific vaccines, such as influenza and pneumococcal vaccination in Greece. In Germany, short clips targeting parents of children are broadcasted in cinemas to promote immunisation against measles. A noteworthy use of educational videos advertised via social media has been implemented in the Netherlands to encourage immunisation against HPV among adolescents and young women [49]. Finally, in Poland, videos have been produced and targeted at pregnant women to increase vaccination against pertussis. 

Ninety per cent of interviewed experts reported websites with educational content implemented in their country, targeting the general population (Greece, Germany, Italy, Malta, the Netherlands, Norway, Poland, Portugal, the United Kingdom). In addition, websites are often specific for some population groups, such as parents of children, adolescents, pregnant women, the elderly, healthcare workers, high-risk groups, and travellers. These websites are hosted and managed by national or regional health institutions, universities, centres for disease control and prevention, and associations of doctors and patients.

In Poland, web portals are hosted by the National Public Health Institute with educational content on mandatory vaccinations with interactive sections that include quizzes.

#### 3.2.5. Other Digital Technologies

Social networks have been reported to be widely employed in all included countries to deliver educational content to the general public in an easy and accessible way [50]. 

On the other hand, none of the interviewed experts recalled the systematic use of automatic telephone calls in immunisation programs in their country. At the same time, many of them suggested that non-automated telephone calls are still the preferred method in vaccination centres at the local level.

In the Netherlands, e-games have been developed to explain the crucial importance of vaccination to children. In Norway, several webinars have been organised to deliver educational content, such as specific campaigns on influenza vaccination for the elderly and HPV for adolescents, young women, and their mothers [51]. 

In most cases, the institutions hosting these interventions are the national authorities and scientific associations of public health physicians.

A promising tool implemented in the UK is electronic consent, which allows parents to fill in an online consent form and let adolescents receive HPV vaccinations in schools [52].

### 3.3. Digital Tools to Support Healthcare Providers in the Delivery of Vaccinations

**Figure 3:**
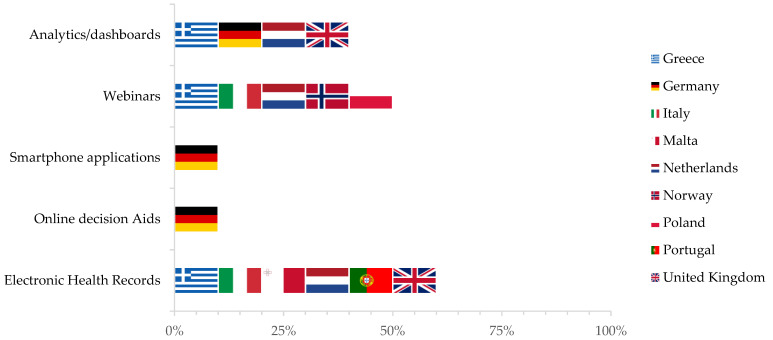
Digital tools used to support healthcare providers in vaccination delivery by country (as reported during in-depth interviews with public health and immunisation experts).

Digital tools can play a crucial role to support healthcare providers in the delivery of vaccinations and have been implemented in 90% of included countries (nine countries: Greece, Germany, Italy, Malta, the Netherlands, Norway, Poland, Portugal, the United Kingdom). Details are provided in Figure 3.

Training webinars on vaccination delivery and the side effects of childhood immunisations are regularly provided to healthcare workers in five included countries (50%). 

In Greece, analytic dashboards are available for pharmacists to monitor vaccination coverage in their jurisdiction, particularly against the flu, while others are currently being developed with data from EHRs. In Germany, with the same purpose, online decision aids, smartphone applications, and dashboards are widely available for general practitioners, physicians in public health services, and paediatricians. In particular, a free smartphone application with push notifications about vaccination recommendations and decision support tools is operational. In Malta and Norway, it is customary for government employees to receive periodic emails with information about available vaccination and encouragement to adhere to these campaigns.

### 3.4. Perceived Challenges and Barriers in the Development of Digital Tools-Based Interventions

**Figure 4:**
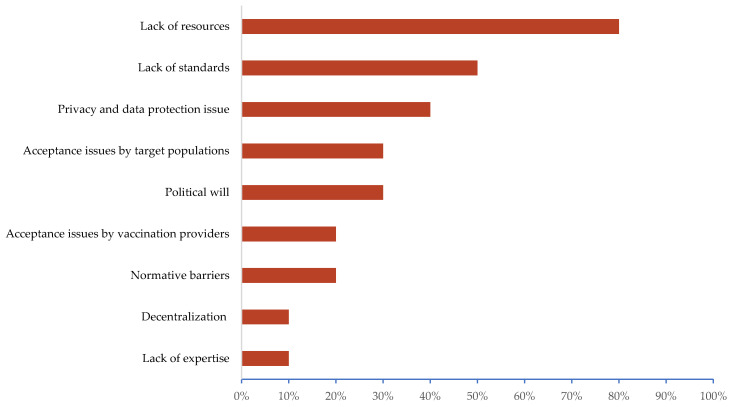
Perceived challenges and barriers to implementing vaccination campaigns based on digital tools, as reported during in-depth interviews with public health and immunisation experts.

Experts identified a lack of resources at the national, regional, or local level as the main barrier (80%, eight countries), followed by a lack of existing and shared standards (50%, 5 countries). Other reported issues were poor acceptance of technologies by healthcare providers (20%), lack of technological literacy of the target populations (30%), and regulatory barriers applicable to this field (20%) (Figure 4).

## 4. Discussion

We conducted a comprehensive assessment on the level of implementation of digital technologies in the context of immunisation programmes in selected European countries. Our results highlighted that, despite 80% of countries reporting digital tools to be used within immunisation programmes, the levels of implementation differ widely among European countries, with some having robust e-Health strategies already in place and functioning, while others are at preliminary stages of implementation. 

As emerges from the data retrieved, national or regional-level EHRs are the tool toward which most interest is directed, given the potential to enable the provision of patient and people-centred care and prevention. EHRs and IISs are key elements to record immunisation data, identify non-immunised subjects, and, eventually, perform timely estimates of vaccination coverages [18,53]. Moreover, interoperable IISs can be a source of data for other ICT interventions, such as text and emails delivering educational content and/or reminders and recalls [54]. However, substantial differences in the level of implementation persist among European countries; thus, further use of new technologies could positively impact immunisations and surveillance data and their quality. Building a robust data culture and integrating existing electronic records is a priority, which should be guided by new evidence on ICT interventions [55].

Despite concerted efforts to promote the development and use of EHRs, websites and educational videos are still the most adopted digital tools in vaccination programs [56]. Effective communication strategies are essential to counteract vaccine hesitancy [57,58]; in this context, such digital tools are used to convey information about the safety and efficacy of vaccines but also on how to access vaccine services [59,60].

The use of digital reminders, automated telephone calls, and email is still limited, and the systems are rarely entirely automated; reminders can include text messages sent to patients to pre-schedule immunisation visits to promote influenza vaccine uptake or immunisation in non-compliant adolescents. Scant quantitative evidence is available to support the superiority of these tools as compared to traditional methods; so, as proposed by Bozzola et al. [16], one option would be to diversify public health campaigns with a hybrid model that would include digital and non-digital interventions, in order to support the inclusion of marginalised populations that may have difficulties in accessing digital resources.

However, we believe the automation of reminder recalls can be a further cornerstone of vaccination interventions in Europe in the future, given the advantages of reduced cost and easier reach of immunisation target populations [28]. Concerning the involvement of target populations in immunisation programs, Atkinson et al., through a systematic review, evaluated the effectiveness of digital push interventions compared to non-digital interventions for specific target populations and high-risk groups. The study highlighted that patients had 1.18 (95% CI 1.11–1.25) greater odds of receiving vaccination or series completion with digital push interventions compared to controls. In parents of children aged 18 and younger, those receiving digital push had a 1.22 (95% CI 1.15–1.30) greater odds than controls [26].

Digital tools can also be essential to support healthcare providers in the delivery of vaccinations and immunisation services. In particular, our study highlights that webinars and dashboards are extensively used to train, re-train, and support health care providers. 

Although our study shows that there are still significant differences in the state of development and use of digital technologies in immunisation programmes, investments in this field can be crucial to develop more efficient public health policies and promote informed preventive behaviours against vaccine-preventable diseases (VPDs). Tozzi et al. focused on identifying the issues and challenges of immunisation programs for which digital tools are potential solutions [61]. Some important overlooked features were (i) the partial automation of procedures that can speed up the process and reduce the workload (e.g., the identification of non-immunised subjects); (ii) digital data sharing and analysis that can help with reporting results (e.g., vaccination coverage estimates); (iii) the integration of multiple data sources, which helps to predict infectious disease epidemiological trends; (iv) direct interaction with target populations, which supports both a precision approach to preventive strategies, as well as people empowerment (e.g., smartphone apps or other tools to deliver personalised educational messages or reminders). 

However, investments and implementation of digitalisation are not without difficulties. Our results shed light on the barriers preventing European countries from implementing digital tools-based vaccination campaigns lacking sufficient resources, standards and common regulatory frameworks. 

Our cross-sectional study was conducted through a structured questionnaire administered in-person by ad hoc trained researchers, which helped us focus on numerous aspects of an extensive topic. It also provided substantial stimuli for discussion with the interviewees, thus allowing the collection of a large amount of qualitative data. We are aware that questionnaires are intrinsically affected by certain limitations: for instance, recall bias and social desirability bias. We tried to limit the effects of these biases in four ways: (i) we shared the questionnaire with the interviewees beforehand, (ii) we administered the questionnaire through structured interviews, (iii) we sent a follow-up email asking that interviewees provide missing details and references about the interventions mentioned during the interview, and (iv) we verified the collected information via a search of grey literature. Lastly, our study did not investigate quantitatively the effectiveness of digital tools used to increase vaccination coverage, and we did not explore the use of EHRs or, specifically, digital immunisation records to monitor adverse events following immunisation (AEFI) in integrated pharmacovigilance surveillance that could have been beneficial for the mass vaccination programmes implemented to face the COVID-19 pandemic. Nevertheless, we collected qualitative data from experts who witnessed the effects of digital-based campaigns in real life. However, the qualitative data collected were not sufficient to provide a comprehensive picture of the effectiveness. Indeed, people we consulted could only provide very scant data on the real-life effectiveness of the digital-based interventions described, as almost no monitoring and evaluation systems were in place to quantitatively estimate the impact of introducing digital components within immunisation programmes, and it was difficult to measure their effects on vaccine uptake rates and other indirect outcomes [62].

To our knowledge, this is one of the first attempts to explore the level of implementation of digital tools to increase vaccination uptake in Europe. Indeed, even though analyses of the use of digital tools in public health campaigns are available in the literature, they are often limited to a single technology; in our research, we broadened the field with an extensive list of digital tools with the aim of providing a comprehensive overview [63]. Future research is needed to investigate the topic further and extend the number of countries included in the analysis. However, reports such as ours are critical as they offer an up-to-date overview, stimulate best practices’ sharing, and inform the planning and implementation of innovative targeted interventions in the field of immunisation practice and research. 

Since this is an expanding field, the status quo in each country and setting can rapidly change, as dramatically shown during the COVID-19 outbreak [64,65]. The need to reorganise healthcare services has led to a massive application of digital interventions (population surveillance, contact tracing, case identification) and highlighted the added value digitalisation could give to public health in the near future, including the delivery of mass-immunisation services [26,64,66]. 

Beyond the undoubted challenges of the process, great interest in this field has been expressed by institutional bodies to innovate and improve vaccination programmes worldwide.

In 2018, the European Observatory on Health Systems and Policies, at the request of the EC, prepared a report on the organisation and delivery of vaccination services in the EU, which included a review of the current situation on vaccine uptake and VPDs and an umbrella review of systematic reviews on health system-related factors influencing vaccine uptake [67]. In the same year, the EC, with a communication on the transformation of digital health and care, identified three main pillars on which the digitalisation process should be centred: (i) secure data access and sharing; (ii) connecting and sharing health data for research and improved health; and (iii) strengthening citizen empowerment and individual care through digital services [30]. The commitment to the digitalisation of public health and health services is also evidenced by the presence of funding opportunities promoted by the EC: EU4Health, Horizon Europe, and some sections of the Digital Europe Program dedicated to healthcare services are aimed at supporting a large-scale deployment of digital solutions within Europe.

An example of an EU Horizon 2020 project is the Models of Child Health Appraised (MOCHA), conducted from 2015 to 2018 in all EU/EEA countries [38]. The study aimed at exploring the level of implementation and the potential role of e-Health in the context of primary and preventive child healthcare and the use of primary care EHR systems, including immunisation data [68]. The project showed that in 2018, 87% of respondent countries only used paper home-based records, with only three countries having plans (Austria, Bulgaria, and Portugal) for digitalisation of health records and four developing patient portals (Denmark, Estonia, Greece, and the Netherlands). Others had unofficial products available, neither validated nor regulated. 

The Regional Office for Europe of the WHO also showed great interest in digitalisation. In the context of the European Programme of Work, 2020–2025—“United Action for Better Health in Europe” (EPW) [69], four flagship initiatives were promoted, two of them addressing digital health empowerment within Europe and the development of a European immunisation agenda 2030. These initiatives will strengthen national immunisation policies and implement service delivery in synergy with global policies, facilitating cross-sector partnerships among regional, subregional, and national institutions. With particular reference to the “empowerment through Digital Health” initiative, the WHO aims to finalise the European Roadmap for Digitalisation of Health Systems and develop a European health data governance framework through a European health data governance charter. The latter will include a set of European values, principles, and methods for health data access, management, governance, and use for effective health systems and public health action.

However, to fully exploit the potential of digitalisation, we believe it is essential to derive recommendations from the analysis of current barriers.

First, the need to enhance political commitment and international alignment of international strategies was frequently reported by interviewed experts. It is necessary to launch cross-border collaboration among European and international institutions responsible for promoting digital health strategies and sharing “best practices”. Adequate political commitment, accompanied by appropriate economic investments, can help reduce the divergence in digitisation strategies between different European countries and create a common framework for the legal, technical, and organisational aspects. 

Efficient use of the technical infrastructures also requires a multi-disciplinary approach to the design, implementation, and monitoring of immunisation programmes so as to count on medical and public health expertise, together with input from engineers, computer scientists, and ICT specialists. Healthcare professionals should be trained to act in a digitalised working environment and interact with professionals whose competencies can boost the impact of digital technologies from a public health perspective. Training of healthcare workers aimed at increasing digital literacy will be fundamental for proper implementation of digital strategies, and elements of digital health should be, in our opinion, included in graduate, post-graduate, or updating programmes.

Last but not least, active involvement of immunisation target populations and promotion of digital health literacy are crucial to ensure accessibility to digital tools and counter vaccine hesitancy [70]. In this regard, our study highlighted significant differences in the level of technological readiness of the population in included countries.

## 5. Conclusions

The presence of countries facing challenges in keeping up with vaccination uptake and countering vaccine hesitancy highlights the need for innovative digital strategies within vaccination programs. We reported the scattered implementation of digital-based interventions to support vaccine delivery across Europe and inadequate monitoring and evaluation of their impact on population health. Therefore, we sought to raise awareness of the need to invest in digital innovation to foster public and preventive health objectives, collect and share successful examples and best practices, and pursue research to identify how to best adapt and scale them up in the broader European context. 

## Figures and Tables

**Table 1:** Characteristics of included countries.

	Greece	Germany	Italy	Luxembourg	Malta	The Netherlands	Norway	Poland	Portugal	United Kingdom
Population ^1^	10,724,599	83,019,213	60,359,546	613,894	493,559	17,282,163	5,383,212	37,972,812	10,276,617	66,647,112
Younger than 15 years	14.33%	13.60%	13%	16.06%	13.68%	15.85%	17.55%	15.36%	13.70%	17.9%
From 15 to 64 years	63.63%	64.86%	64%	69.55	67.65%	64.97%	65.21%	66.98%	64.47%	63.7%
65 years or over	22.04%	21.54%	23%	14.39%	18.68%	19.18%	17.24%	17.66%	21.84%	18.4%
Socio economic indicators ^1^										
Real GDP per capita (2019)	18,150	35,840	28,860	83,640	22,040	41,870	69,870	12,980	18,540	32,980
Unemployment rate (2019)	17.3%	3.2%	10%	5.6%	3.4%	3.4%	3.35%	3.3%	6.5%	3.8%
Population (15–64 years) with tertiary education (ISCED 2011 levels 5–8)	27.8%	25.9%	17.4%	41%	26.1%	34.8%	37.7%	28.2%	23.8%	40.6%
Vaccination Coverage										
DTP1 (2019) ^2^	99%	99%	95%	99%	98%	n.a.	99%	n.a.	n.a.	n.a.
MCV1 (2019) ^2^	97%	97%	94%	99%	96%	94%	97%	93%	99%	91%
Influenza (2017) ^3^	n.a.	34.8%	52%	n.a.	n.a.	64.1%	26.9%	6.87%	60%	n.a.
Indicators of digitalization ^1^										
Household-level internet access	79%	95%	85%	95%	86%	98%	98%	87%	81%	96%
Individuals’ internet use	76%	94%	78%	97%	78%	96%	99%	82%	76%	96%
Digital inclusion of individuals	74%	91%	74%	93%	85%	95%	98%	78%	73%	95%
E-government activities of individuals, via websites	52%	59%	23%	60%	50%	81%	87%	40%	41%	63%
Individuals who have basic or above basic overall digital skills	79%	70%	42%	65%	42%	79%	83%	44%	52%	74%

DTP1: diphtheria, pertussis and tetanus first dose of vaccine; MCV1: measles-containing vaccine first dose; n.a.: not available; ^1^ Eurostat online database accessed on 30 July 2020 (https://ec.europa.eu/eurostat/data/database); ^2^ WHO report on vaccination coverage, accessed on the 30 July 2020 (https://www.who.int/immunization/monitoring_surveillance/data/en/); ^3^ ECDC Seasonal influenza vaccination and antiviral use in EU/EEA Member States accessed on 30 July 2020 (https://www.ecdc.europa.eu/en/publications-data/seasonal-influenza-vaccination-antiviral-use-eu-eea-member-states).
